# The RUNX1/RUNX1T1 network: translating insights into therapeutic options

**DOI:** 10.1016/j.exphem.2020.11.005

**Published:** 2021-02

**Authors:** Laura E. Swart, Olaf Heidenreich

**Affiliations:** Princess Maxima Center for Pediatric Oncology, Utrecht, The Netherlands

## Abstract

*RUNX1/RUNX1T1* is the most common fusion gene found in acute myeloid leukemia. Seminal contributions by many different research groups have revealed a complex regulatory network promoting leukemic self-renewal and propagation. Perturbation of RUNX1/RUNX1T1 levels and its DNA binding affects chromatin accessibility and transcription factor occupation at multiple gene loci associated with changes in gene expression levels. Exploration of this transcriptional program by targeted RNAi screens uncovered a crucial role of RUNX1/RUNX1T1 in cell cycle progression by regulating CCND2. This dependency results in a high vulnerability toward inhibitors of CDK4 and CDK6 and suggests new avenues for therapeutic intervention against acute myeloid leukemia.

## Highlights

• A RUNX1/RUNX1T1-associated gene expression network drives acute myeloid leukemia.
• RUNX1/RUNX1T1 dysregulates gene expression at multiple levels.
• Interrogation of the RUNX1/RUNX1T1 network by RNA interference or CRISPR screens identifies novel therapeutic targets.
• RUNX1/RUNX1T1 drives cell cycle progression in G1 by promoting CCND2 and CDK6 expression.
• RUNX11/RUNX1T1-positive AMLs are sensitive toward CDK4/6 inhibitors.

Fusion genes and their underlying chromosomal alterations are a hallmark of acute myeloid leukemia (AML). They are particularly prevalent in pediatric AML, where more than 50% of all cases harbor a fusion gene [1,2]. As fusion genes predict clinical outcome, they are used for patient stratification in the World Health Organization (WHO) 2016 classification of AML [3]. More than 60% of all rearrangements found in pediatric AML target only five different protein complexes: the core binding factor (CBF), the epigenetic regulator MLL, the nuclear receptor RARA, and the nuclear pore component NUP98 [1,2]. Interestingly, all five complexes including NUP98 directly regulate transcription. The rearrangements replace a functional, transactivating or repressing domain with parts of the second fusion partner, thereby generating transcriptional modulators with novel functionalities.

Most of these translocations are initiating events in the process of leukemogenesis and are expressed in all leukemic cells of a patient. The generation of fully transformed leukemic cells usually requires the acquisition of several mutations that act in concert to drive self-renewal and proliferation, sustain viability, and avoid differentiation and immune surveillance [4]. Classically, these mutations are divided into two classes. While class 1 comprises mutated kinase genes such as *BCR/ABL1* or *FLT3-ITD* and is thought to promote proliferation and survival, fusion genes encoding transcription factors such as *MLL/AF9, PML/RARA* or *RUNX1/RUNX1T1* (*AML1/ETO, AML1/MTG8*) have been classified as type 2 mutations that drive self-renewal and interfere with differentiation. Recently, a third class has been suggested that separates mutated epigenetic modifiers and readers from class 2. However, more recent investigations of transcriptional networks underlying the leukemic phenotypes have blurred this clear distinction, with type 2 members also promoting proliferation by controlling growth factor and cell cycle genes such as *FLT3, CDK6*, and *CCND2* 5, 6, 7. As preleukemic and leukemic self-renewal is dependent on cell cycle progression, it is not really surprising that class 2 mutations also drive proliferation as part of their program.

Importantly, perturbation studies have revealed that human leukemic cells are dependent on continuing expression of their respective type 2 fusion gene, with its transient loss leading to impaired proliferation and increased senescence [8]. This dependence of leukemia on continuing expression of fusion genes is based on the leukemic programs and their underlying transcriptional networks linked to the corresponding fusion protein. Fusion proteins such as RUNX1/RUNX1T1 and CBFB/MYH11 are linked by unique transcription factor networks and are subject to posttranslational modifications regulating their activities [9]. These networks are composed of nodes common to all AML types such as the AP-1 and Krüppel-like factor (KLF) nodes and those being characteristic of a given AML subtype such as the HOXA, NFIL3, and POU4F1 nodes. For instance, the HOXA node separates AML into two classes: those that activate the *HOXA* cluster (e.g., AMLs with *MLL* or *NUP98* rearrangements) and those that do not (e.g., AMLs with *RARA* or core binding factor rearrangements) [9,10]. Thus, the different fusion proteins employ distinct transcription factor networks to establish and drive the corresponding AML subtype.

## Dissecting transcriptional programs of leukemic fusion proteins

Because of their biological significance and exclusive expression in preleukemic and leukemic cells, fusion genes are highly attractive targets for any precision medicine approach. However, their direct targeting by more conventional drug discovery approaches, such as small molecules and monoclonal antibodies, has proven to be challenging, CBFB/MYH11 being so far the only example of a truly fusion protein-specific targeting [11,12]. An alternative way to specifically target fusion genes is by RNA interference (RNAi)–based approaches. RNAi is a naturally occurring mechanism that allows sequence-specific gene silencing. Discovery of this pathway opened the way for many, previously, unreachable targets as RNAi can be triggered by exogenous introduction of dsRNA or constructs [13]. This approach, however, requires complex lipid or polymeric formulations for efficient drug delivery because of the unfavorable pharmacokinetic (PK) properties of siRNAs 14, 15, 16.

Alternatively, the leukemic programs and transcriptional networks associated with fusion gene-encoded transcriptional regulators offer new options for targeted therapeutic interventions. Pathways under the direct control of the corresponding fusion protein are likely to contain elements amenable to pharmacologic interference. The challenge here is to have sufficient insight into the composition of these fusion protein-driven programs and the relevance of its components for leukemic maintenance. These programs can be unraveled by fusion protein knockdown or inhibition of its function, followed by analysis of changes in chromatin occupation and gene expression. Integration of RNA-seq, ChIP-seq, and DHS-seq data revealed the compositions of several AML networks including those for RUNX1/RUNX1T1, CBFB/MYH9, and CBFA2T3/GLIS2 [11,17,18]. Compared with the whole genome or transcriptome, such well-characterized networks have a substantially lower complexity, thus facilitating further functional characterization by targeted RNAi or CRISPR screens.

These transcription factor nodes add additional levels of complexity with respect to the transcriptional regulation of the whole network. Fusion proteins may affect transcription directly by physical interaction with promoters, enhancers, or silencers, indirectly via regulation of expression and activity of transcription factors, or even in a mixed mode involving both direct binding of the fusion protein and recruitment of downstream transcription factors, consequently exacerbating the identification of direct target genes. Generally, direct target genes are defined by changes in transcript levels upon perturbation of the transcription factor in combination with binding of the transcription factor to the gene locus or to a more distant regulatory element such as an enhancer or silencer [6]. Consequently, integration of three-dimensional interaction analyses provides more detailed insights into fusion protein-exerted control of transcriptional networks and a more comprehensive list of direct target genes [19].

## The RUNX1/RUNX1T1 interactome

**Figure 1:**
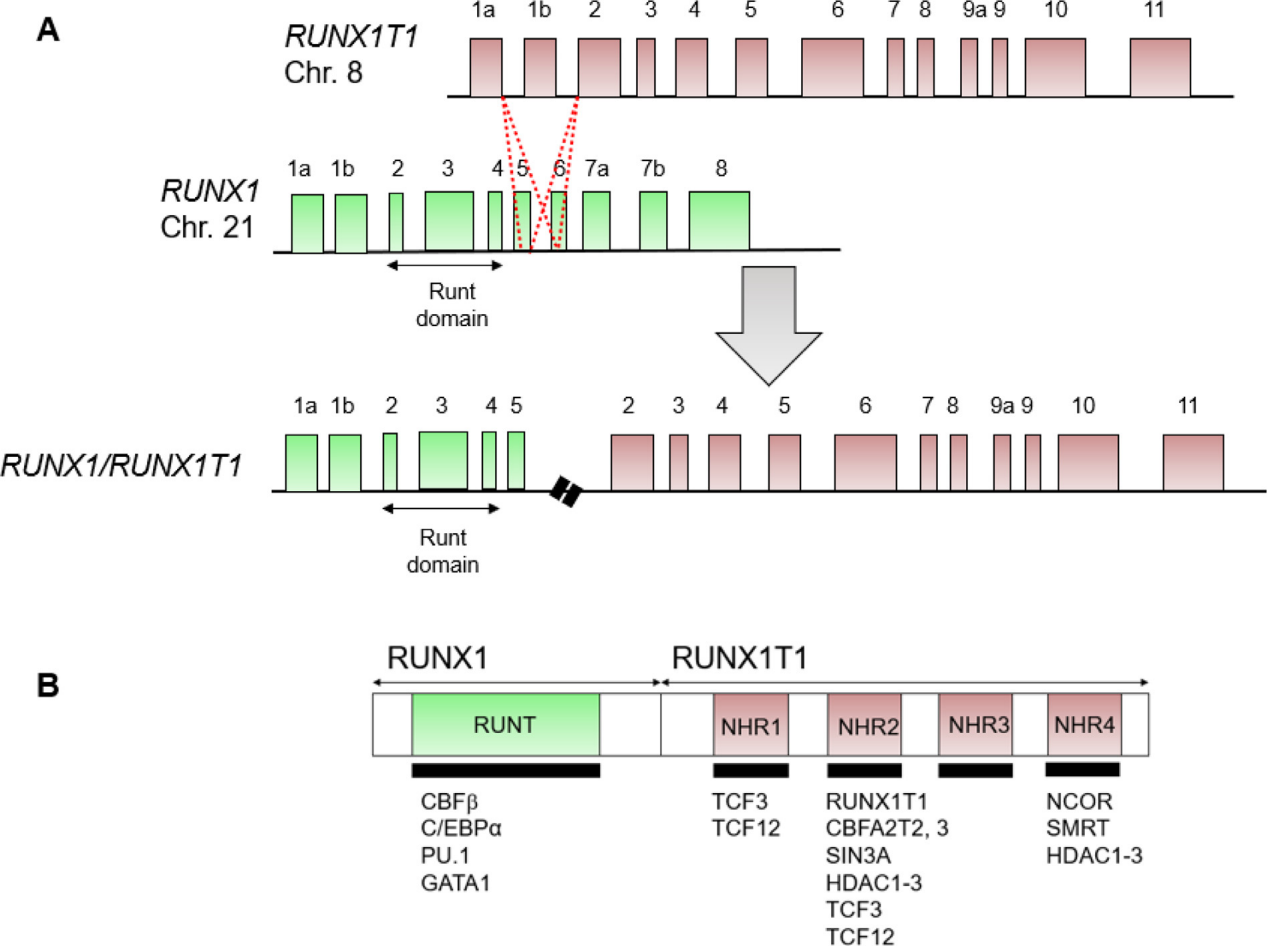
Structure of the RUNX1/RUNX1T1 fusion gene and protein. **(A)** Organization of the fusion gene. The breakpoints cluster within RUNX1T1 exon 1a–2 and RUNX1 exon 5–6. **(B)** Structure of the fusion protein. Transcription factors and epigenetic regulators that are forming complexes with RUNX1/RUNX1T1 are indicated below the regions with which they are interacting.

The chromosomal translocation t(8;21)(q22;q22), generating the *RUNX1/RUNX1T1* fusion gene, is the most prevalent chromosomal rearrangement, with an incidence of 15% in children and young adults [20]. While RUNX1 is a key transcription factor in the early stages of hematopoiesis [21], the role of RUNX1T1 (ETO, MTG8, CBFA2T1) is less well understood [22]. Like its other family members, RUNX1T1 is a transcriptional co-repressor [23,24]. In contrast to its paralogues, RUNX1T1 is only more prominently expressed in the megakaryocytic and erythrocytic lineages, basophiles and eosinophiles, and B progenitors. It is, however, hardly expressed in the hematopoietic stem and progenitor cell (HSPC) compartment. The translocation juxtaposes the DNA-binding Runt domain of RUNX1 and the almost complete open reading frame of RUNX1T1, with chromosomal breakpoints clustering within RUNX1 intron 5 and RUNX1T1 intron 1 (Figure 1A) [25,26]. A paralogue of RUNX1/RUNX1T1, CBFA2T3 is part of an alternative fusion with RUNX1 in the translocation t(16;21)(q24;q22) associated with a phenotypically very similar AML [27].

The transcriptional network established and maintained by RUNX1/RUNX1T1 is one of the best characterized leukemic networks. RUNX1/RUNX1T1-exerted control is modulated by several posttranslational modifications including acetylation and ubiquitylation by EP300 and UBE2L6, respectively, and depends on the recruitment of epigenetic modifiers, in particular class I histone deacetylases via SIN3A and NCOR via its RUNX1T1 moiety (Figure 1B) 28, 29, 30, 31, 32, 33, 34.

Similar to wild-type RUNX1, RUNX1/RUNX1T1 heterodimerizes with CBFβ, increasing its DNA binding affinity, although the relevance of this interaction for the function of RUNX1/RUNX1T1 has remained a matter of debate [35,36]. Furthermore, several hematopoietic transcription factors, including GATA1, CEBPA, and PU.1, have been found to interact with the Runt domain of both wild-type and fusion protein [37]. RUNX1/RUNX1T1 forms a complex consisting of CBFβ, E proteins TCF3 and TCF12, LYL1, and bridging factors LMO2 and LDB1 and further cooperates with the ETS family members FLI1 and ERG [38]. In particular, the interaction with E proteins has been investigated in greater detail, where RUNX1/RUNX1T1 binds them through its NHR1 and NHR2 domains. Interestingly, this interaction prevents recruitment of p300/CBF co-activators [39,40]. Nevertheless, p300 has been reported to modulate RUNX1/RUNX1T1 activity by acetylating several N-terminal lysine residues [33].

**Figure 2:**
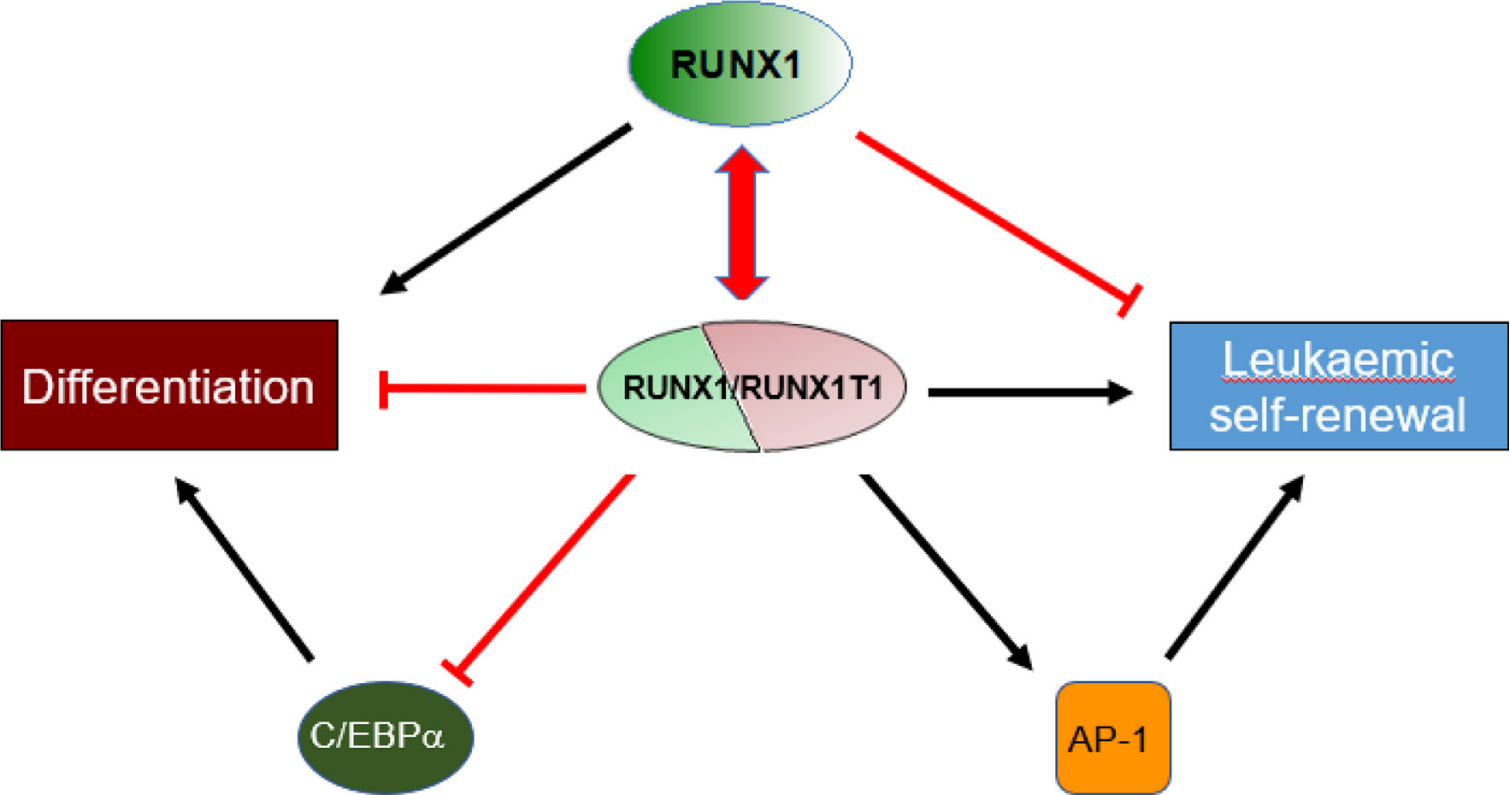
Dynamic interplay between RUNX1 and RUNX1/ETO binding partners. An equilibrium between RUNX1/ETO and RUNX1 is required for leukemic propagation. Repression of C/EBPα and activation of expression of AP-1 subunits impairs myeloid differentiation and promotes leukemic self-renewal and expansion.

A main feature of RUNX1/RUNX1T1-mediated dysregulation of gene expression is its competition with wild-type RUNX1 binding. RUNX1/RUNX1T1 shares with RUNX1 about 75% of its binding sites. A basal level of RUNX1 is required for RUNX1/RUNX1T1 to maintain cell growth, and complete suppression of the differentiation genes is not tolerated [41]. This competition also results in the recruitment protein complexes with opposing activities (Figure 2). For instance, re-chromatin immunoprecipitation (ChIP) experiments revealed that RUNX1/RUNX1T1 recruits HDACs, while RUNX1 cooperates with EP300 on genes repressed by the fusion protein [42]. Both complexes can bind to the same loci, suggesting either differential occupation of alleles or co-occupation on the same allele on sites with multiple binding sites [43]. Furthermore, while RUNX1/RUNX1T1 has been found to interact with DNA methylases DNMT1 and DNMT3A, RUNX1 is able to recruit TET histone demethylases, highlighting again opposing activities associated with wild-type and mutated protein [44,45].

Loss of RUNX1/RUNX1T1 severely compromises malignant self-renewal, but hardly affects viability of t(8;21) AML cells, while depletion of RUNX1 induces apoptosis in this leukemic context [46,47]. This finding suggests that this leukemia is dependent on a balance between RUNX1/RUNX1T1 and RUNX1 and agrees with the clinical observation that mutations in the nontranslocated RUNX1 allele are only rarely found in t(8;21) AML. In addition, positive feedback loops comprise FOXO1, ERG, and AP-1, whose expression is increased by RUNX1/RUNX1T1 and which co-occupy sites and cooperate with RUNX1/RUNXT1T in driving leukemic propagation (Figure 2) [19,48,49]. In contrast, C/EBPα and RUNX1/RUNX1T1 form a negative feedback loop, with the latter directly interfering with the expression of the former [37,42]. Increased expression of differentiation-associated genes is associated with loss of RUNX1/RUNX1T1 binding and dependent on concomitant binding of CEBPα to these sites [42]. Consequently, RUNX1/RUNXT1 employs a complex network of checks and balances comprising binding site competition and feedback loops for establishing and maintaining a leukemic transcriptional program.

## Control of gene expression by RUNX1/RUNX1T1: transcription and beyond

Depletion of RUNX1/RUNX1T1 by fusion site-specific siRNAs has provided some fundamental insights into the transcriptomic program driven by this fusion protein [42,50]. RUNX1/RUNX1T1 knockdown in t(8;21) AML cells causes a significant change in expression of about 2,600 of 15,000 expressed genes showing that RUNX1/RUNX1T1 controls 17% of the whole transcriptome.

Consequently, RUNX1/RUNX1T1 reprograms a massive transcriptional network to establish and maintain leukemia. In agreement with repressing gene function by recruiting class 1 HDACs, RUNX1/RUNX1T1 impairs expression of a set of about 1,400 genes that is linked to myeloid and, in particular, neutrophil differentiation. However, although RUNX1/RUNX1T1 is frequently described as a transcriptional repressor, the vast majority of these genes are rather modestly downregulated, suggesting that a basal expression of these “repressed” genes may not only be tolerated, but actually may be required for the leukemic phenotype. Furthermore, almost 50% of all genes affected are maintained or activated by RUNX1/ETO. This upregulated gene expression signature is associated with several “Hallmarks of Cancer” processes including cell cycle progression at multiple stages, glycolysis and oxidative phosphorylation, MTOR signalling, RNA processing, and ribosome biogenesis [42,50].

In particular, the latter two processes emphasize additional mechanisms of gene expression control exerted by leukemic fusion proteins. RUNX1/RUNX1T1 regulates the generation of alternative transcripts both by affecting alternative splicing and by repressing the activity of alternative promoters of genes such as *RPS6KA1* or *PARL*, thus adding an additional layer of complexity to the RUNX1/RUNX1T1 transcriptional network [51]. Furthermore, and similar to genetic deletion of Runx1 in murine HSCs, RUNX1/RUNX1T1 depletion reduces the expression of multiple ribosomal proteins and has been found to impair the expression of rRNAs by binding together with the RNA POL I factor UBF1 to rDNA repeats [42,52,53]. In line with these findings, the leukemic potential of AML1/ETO9a, a C-terminally truncated isoform of RUNX1/RUNX1T1, depends on AES-driven induction of snoRNAs that control 2′-O-methylation of rRNAs [54]. However, it remains unclear how these changes in expression affect ribosome biogenesis, as knockdown of CBFB/MYH11 caused an increase of polysomes despite impaired rRNA transcription [55].

**Figure 3:**
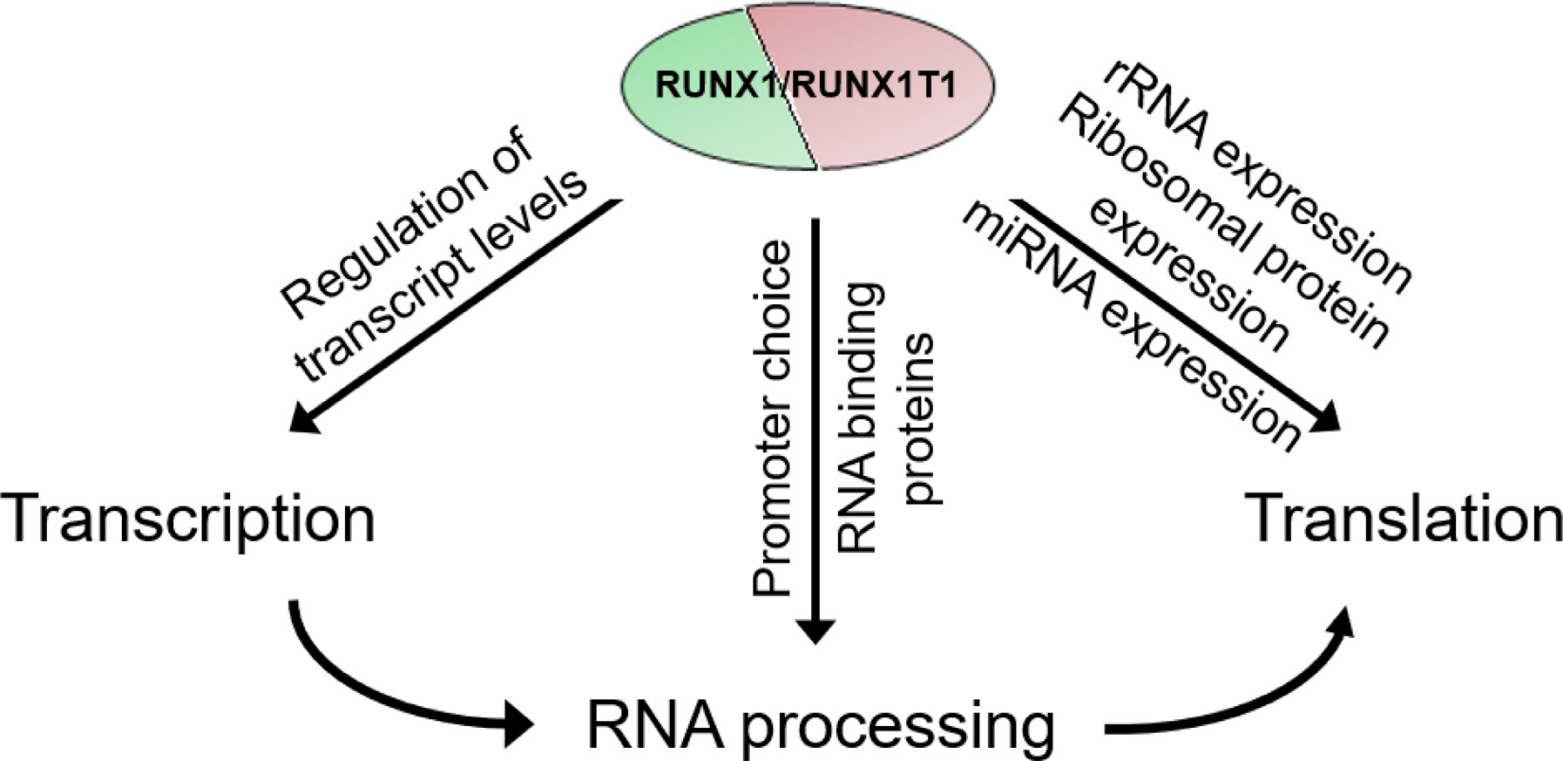
RUNX1/RUNX1T1 regulates gene expression at multiple levels. The fusion protein can dysregulate transcription by binding to promoter, enhancer, or silencer elements. RUNX1/RUNX1T1 also affects the ratios of RNA isoforms by regulating alternative promotor activity and affecting RNA splicing. Lastly, RUNX1/RUNX1T1 has an impact on translation by controlling miRNA and rRNA transcription and ribosomal protein expression.

Finally, RUNX1/RUNX1T1 also controls mRNA translation and stability by perturbing the expression of multiple miRNAs. Genome-wide analysis of AML patient samples revealed that t(8;21) patients have a distinct miRNA signature compared with other chromosomal translocations [56]. For instance, *MIR126*, which reduces apoptosis and increases proliferation, is overexpressed in CBF AMLs [57]. It is an intronic miRNA located in the *EGFL7* gene, a direct target gene of RUNX1/RUNX1T1. Furthermore, repression of *MIR223* by RUNX1/RUNX1T1 impairs granulocytic differentiation and activation of leukemic cells and may also support cell cycle progression by enhanced expression of the *MIR223* target E2F1 [56,58]. These combined findings establish a model in which RUNX1/RUNX1T1 interferes with gene expression at multiple layers: by dysregulation of Pol I and Pol II transcription, by affecting RNA splicing and by interfering with translation via miRNAs and ribosome biogenesis (Figure 3).

## Dissecting the RUNX1/RUNX1T1 network

This large and complex RUNX1/RUNX1T1 network offers both challenges and opportunities for precision medicine approaches. On the one hand, it is predicted to comprise multiple potential targets amenable to conventional drug discovery and development and also a number of candidates for which already clinically approved drugs or at least experimental substances at a preclinical stage are available. On the other hand, because its high complexity, it is challenging to identify those crucial targets whose modulation will lead to a collapse of the net and a potential therapeutic effect. Promising approaches here are RNA interference and CRISPR screens for the functional identification of network components.

To identify crucial components of the leukemic network, we assumed that interference with target genes of RUNX1/RUNX1T1 is less likely to be compensated by altered expression of alternative genes than that of indirect target genes. Direct target genes were selected according to the “guilt by proximity” principle by choosing genes with detectable RUNX1/RUNX1T1 binding within or in the neighborhood of their loci. This approach is likely to underestimate the direct impact of a fusion protein, as distances between gene loci and their regulatory elements may span several hundred kilobases. Furthermore, we focussed on genes whose expression was significantly downregulated by RUNX1/RUNX1T1 knockdown because of their potential involvement in leukemic self-renewal.

Integration of RNA-seq, ChIP-seq, and DNase I hypersensitive site (DHS)–seq experiments identified a set of 133 genes [6]. To minimize counterselection during the enrichment process, a doxycycline-inducible expression system was chosen, where the siRNA sequences were embedded in a MIR30 backbone. The subsequent RNAi screen examined the relevance of these putative self-renewal genes both in tissue culture using serial replating and long-term culture and in vivo after orthotopic transplantation of immunodeficient mice.

This study provided several key findings. Firstly, the in vivo arm provided many more candidates than the in vitro assays, a result that was also described in a similar RNAi screen in glioma [59]. A possible explanation for this difference is that propagation in vivo requires additional functionalities compared with growth in tissue culture because of, for example, interaction with stroma, migration and homing in new sites, and metabolic restrictions. Secondly, the only gene that robustly scored in all in vitro and in vivo arms of the screen was *CCND2*. Validation experiments demonstrated a strict dependency of leukemic clonogenicity and competitiveness on CCND2 expression [6]. Notably, depletion of either RUNX1/RUNX1 or CCND2, as well as pharmacologic inhibition of CDK6, led to senescence even in cell lines harboring mutated p53, suggesting a p53-independent mechanism. Thirdly, RUNX1/RUNX1T1 was also found to bind to an intragenic element in the *CDK6* locus, and its loss was associated with reduced *CDK6* expression. Finally, RUNX1/RUNX1T1-positive AML cell lines and patient cells are very sensitive toward the CDK4/6 inhibitor Palbociclib, exhibiting half-maximal responses at nanomolar concentrations. Thus, RUNX1/RUNX1T1 drives cell cycle progression through the G1 phase by promoting the expression of both components of the CDK6–CCND2 kinase complex*.*

Independent confirmation of the significance of CCND2 came from the Cancer Dependency Map project, where genome-wide RNAi screens identified CCND2 as an essential gene in the two 2 RUNX1/RUNX1T-expressing AML cell lines within a total of 501 cancer cell lines [60]. Moreover, several studies identified CCND2 mutations in 3%–12% of all t(8;21) AMLs, with the majority of them causing an increase in CCND2 protein stability 61, 62, 63, 64, 65, 66. These findings again highlight an dependence of t(8;21) AMLs on CCND2 activity and suggests a genetic manifestation and enhancement of the RUNX1/RUNX1T1-driven expression of CCND2. Similarly, CCND1 was mutated in about 2% of the t(8;21) cases examined, while *CCND3* mutations were found in *MLL*-rearranged AMLs, but not in t(8;21) AML. Although RUNX1/RUNX1T1 occupies the loci of all three D-cyclin genes, RUNX1/RUNX1T1 knockdown reduced *CCND1* and *CCND2*, but not *CCND3* transcript levels [42]. *CCND1* is at least tenfold lower expressed than *CCND2* across AML subtypes and neither scored in RNAi screens nor compensated for loss of CCND2 in t(8;21) cells [6]. These data suggest non-overlapping functions of different D-cyclins in the leukemic context.

It is also interesting to note that RUNX1/RUNX1T1 upregulates *CDKN1A* (*p21*) in a p53-independent fashion, thereby interfering with the CDK2-mediated progression from the G1 into the S phase of the cell cycle [67,68]. It has been suggested that bypassing p21 functionality is one mechanism by which RUNX1/RUNX1T1 induces and drives leukemogenesis [68], with the activation of *CCND2* expression possibly contributing to it. However, CDKN1A has also been reported to support DNA repair and self-renewal of leukemia stem cells upon DNA damage [69]. These on first sight contradictory observations suggest a model in which RUNX1/RUNX1T1 exerts a balanced regulation of cell cycle progression by integrating leukemic proliferation with the necessity to maintain genomic integrity.

Furthermore, the activation of direct target genes by RUNX1/RUNX1T1 seems to be surprising, given that the translocation replaces the RUNX1 transactivation domain by several repressor domains of RUNX1T1. However, detailed analysis of the impact of RUNX1/RUNX1T1 depletion on chromatin accessibility, histone modifications, and transcription factor occupation of the *CCND2* locus revealed that RUNX1/RUNX1T1 binds mainly to an element 30 kb upstream of CCND2’s transcriptional start site (TSS) [6,19]. Hi-C chromatin conformation capture analysis indicated that both elements interact with each other and that loss of RUNX1/RUNX1T1 leads to a strengthening of this interaction. These findings suggest that RUNX1/RUNX1T1 supports *CCND2* expression by modulating a three-dimensional interaction between its promoter region and a silencing element. Interestingly, the histone acetyl transferase EP300 binds to the promoter region of *CCND2*. As EP300-mediated acetylation of lysines located at the N-terminus of RUNX1/RUNX1T1 can support its self-renewal function, it is tempting to speculate that this modification may be involved in the activation of *CCND2* transcription, similar to what has been described for other upregulated genes such as ID1 [33].

In addition, RUNX1/RUNX1T1 also maintained *CCND2* transcription indirectly via the JUN-FOS heterodimer AP-1. Knockdown of RUNX1/RUNX1T1 reduces expression of both FOS and JUN members and blocks JUND binding to the *CCND2* promoter. In line with these findings, expression of transdominant negative FOS, which interferes with all AP-1 isoforms, reduces CCND2 transcript levels and severely impairs proliferation of the RUNX1/RUNX1T1-expressing AML cells [9]. These combined data suggest a mixed-type regulation model for *CCND2*, where loss of RUNX1/RUNX1T1 directly changes chromatin accessibility and the three-dimensional communication between promoter and distal elements, and impairs chromatin occupation by AP-1 members.

## Therapeutic targeting of the cell cycle in AML

**Figure 4:**
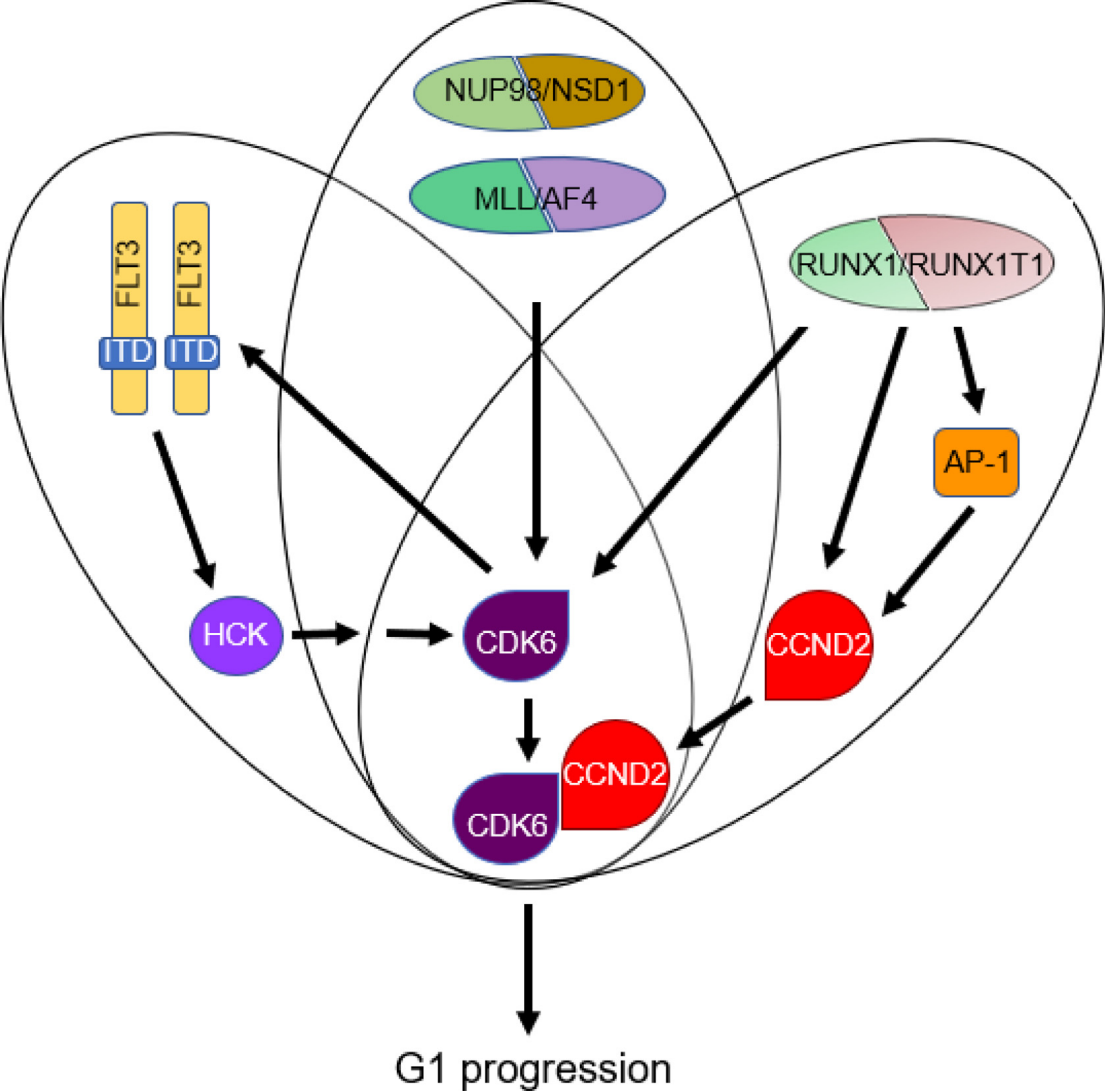
Dysregulation of CDK6 by leukemic mutations. Leukemic fusion proteins such as MLL/AF4, NUP98/NSD1, and RUNX1/ETO either directly drive CDK6 expression or, in the case of RUNX1/ETO, enhance expression of CCND2. FLT3-ITD forms with CDK6 a positive feedback loop, where FLT3-ITD activates CDK6 expression via the SRC kinase HCK, while its own transcription is driven by CDK6.

Facilitating cell cycle progression by supporting expression and function of CDK6 or D-cyclins has also been observed for other fusion genes and therewith associated mutations. Several functional studies, including those employing RNAi screens, have highlighted dependence of acute leukemias to expression of CDK6 or D-cyclins [6,70, 71, 72]. Many of the corresponding gene loci are bound by leukemic fusion proteins and may, thus, be directly controlled by them. For instance, the*CDK6* and *CCND3* loci are direct target genes of MLL/AF4, the most frequent fusion protein found in infant leukemia, and several NUP98 fusion proteins drive *CDK6* transcription by binding to its promoter [5,7]. Thus, promotion of G1 cell cycle phase progression by activation of *CDK6* expression is a common theme for leukemic fusion proteins (Figure 4).

The CDK6/CCND complex is also targeted by several kinases that are recurrently mutated in AML. In particular, FLT3-ITD has been found to be linked to CDK6 in a reciprocal manner. On the one hand, CDK6 binds to the *FLT3* locus and drives its expression [71]. On the other hand, FLT3-ITD increases *CDK6* transcription by activating the Src kinase HCK [73]. This positive feedback loop offers exciting opportunities for drug combinations to combat drug resistance. In this context it is interesting to see that FLT3-ITD frequently co-occurs with the NUP98/NSD1 fusion protein, suggesting a scenario in which two mutations may synergize to drive *CDK6* expression and cell cycle progression [10].

These combined data indicate that a significant portion of AMLs are addicted to CDK6 activity, which creates new therapeutic opportunities. The availability of several clinically approved CDK4/6 inhibitors offers new opportunities for translating the addiction of acute leukemias on G1 CDK activity into treatment strategies. Currently, the European Medicines agency (EMA) and the U.S. Food and Drug Administration (FDA) have approved palbociclib, ribociclib, and abemaciclib, three different compounds with similar activities and properties, for the treatment of breast cancer. In addition, nine trials testing palbociclib or ribociclib in acute leukemias are listed in the ClinicalTrials.gov database. This number does not consider trials such as the European Proof-of-Concept Therapeutic Stratification Trial (ESMART), in which leukemias are part of the tumour portfolio examined. Recent data suggest that all three inhibitors do not directly inhibit CDK4/6 activity but prevent the association of the CDKs with their CCND partners [74]. This conclusion is also supported by a comparison of gene expression patterns by gene set enrichment analysis (GSEA), which revealed a high correlation between CCND2 knockdown and palbociclib treatment in t(8;21) cells [6].

However, high CCND2 expression is associated with a better clinical outcome in AML, suggesting that CCND2 may contribute to the relatively high chemosensitivity of t(8;21) AML. Interestingly, although neither *CDK4, CDK6*, or *CCND1* predict outcome, high levels of*CCND3* indicate worse outcome. The reason for this is currently unknown but may possibly reflect distinct substrate preferences of the different CDK–CCND complexes.

Because of their cytostatic action, CDK4/6 inhibitors will be ineffective as single agents and, thus, need to be applied as part of combination treatments with targeted compounds such as MTOR inhibitors or as part of a scheduled regimen with classic chemotherapeutics [75]. Classically seen as a driver of cell cycle progression by phosphorylating and inactivating RB1, recent findings point to a much more complex role by coordinating multiple cellular processes ranging from metabolism over DNA integrity to antigen processing and presentation 76, 77, 78, 79, 80, 81, 82, 83. Given this complexity it may not be a surprise that leukemic fusion proteins do target such central cellular relay stations. Future research will indicate whether and how this intimate relationship can be therapeutically exploited.

## Conflict of interest disclosure

The authors declare no conflicts of interest.
